# Supplementary material for: The causal relationship between the human gut microbiota and pyogenic arthritis: a Mendelian randomization study
Source: Front Cell Infect Microbiol. 2024 Nov 26;14:1452480. doi: 10.3389/fcimb.2024.1452480 (PMC11629706; doi:10.3389/fcimb.2024.1452480)
Supplement: Supplementary file 1 [file DataSheet1.zip › mendelian test/ebi-a-GCST90027729.csv_leave_one_out.pdf]

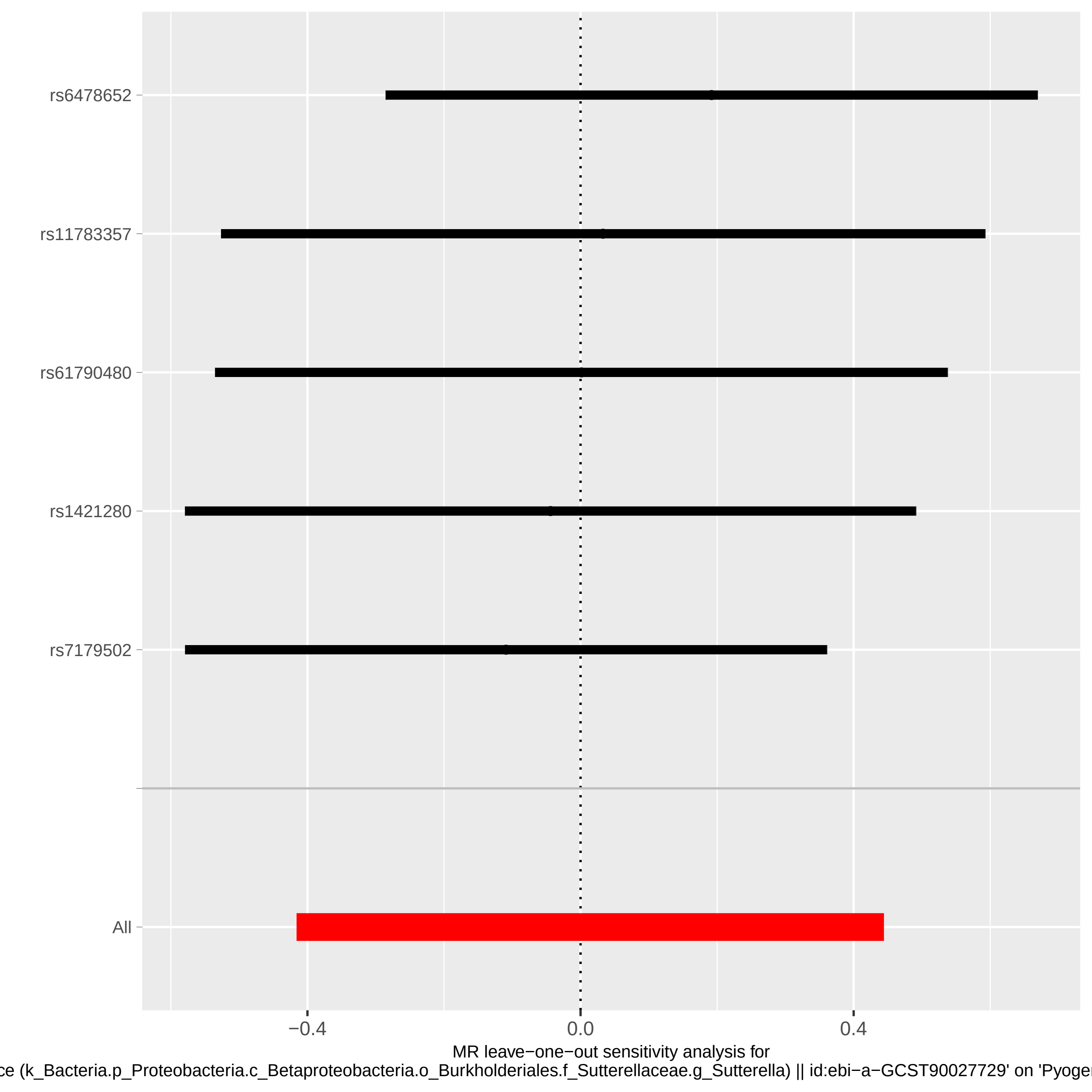
rs6478652
rs11783357
rs61790480
rs1421280
rs7179502
All
−0.4
0.0
0.4
MR leave−one−out sensitivity analysis for
ce (k_Bacteria.p_Proteobacteria.c_Betaproteobacteria.o_Burkholderiales.f_Sutterellaceae.g_Sutterella) || id:ebi−a−GCST90027729' on 'Pyoge
